# Hsa_circ_0003159 inhibits gastric cancer progression by regulating miR-223-3p/NDRG1 axis

**DOI:** 10.1186/s12935-020-1119-0

**Published:** 2020-02-19

**Authors:** Jingyu Wang, Weize Lv, Zhidong Lin, Xiao Wang, Juyuan Bu, Yonghui Su

**Affiliations:** 1 Department of Gastrointestinal Surgery, The Fifth Affiliated Hospital of Sun Yat-Sen University, No. 52, East Meihua Road, Zhuhai, 519000 Guangdong China; 2 Department of Thoracic Oncology, The Fifth Affiliated Hospital of Sun Yat-Sen University, Zhuhai, China; 3 Department of General Surgery, The Fifth Affiliated Hospital of Sun Yat-Sen University, Zhuhai, China

**Keywords:** Gastric cancer, hsa_circ_0003159, miR-223-3p, NDRG1, Proliferation, Migration

## Abstract

**Background:**

Abnormally expressed circular RNAs (circRNAs) are implicated in the development and treatment of gastric cancer (GC). Previous study has reported that hsa_circ_0003159 is expressed in GC. However, the role and mechanism of hsa_circ_0003159 in GC progression remain unclear.

**Methods:**

GC tissues and normal tissues were harvested from 55 patients in this study. The levels of hsa_circ_0003159, microRNA (miR)-223-3p and N-myc downstream regulated gene 1 (NDRG1) were measured by quantitative real-time polymerase chain reaction or western blot. Cell proliferation, migration, invasion and apoptosis were determined by cell counting kit (CCK)-8, transwell assay, flow cytometry and western blot, respectively. The target association of miR-223-3p-hsa_circ_0003159 and miR-223-3p-NDRG1 was explored by dual-luciferase reporter assay. Xenograft model was established to assess the roles of hsa_circ_0003159 in GC in vivo.

**Results:**

Hsa_circ_0003159 was lowly expressed in GC tissues and cells and mainly presented in the cytoplasm. Low expression of hsa_circ_0003159 was associated with lower overall survival and disease-free survival. Hsa_circ_0003159 overexpression inhibited proliferation, migration and invasion but induced apoptosis in GC cells. MiR-223-3p was a target of hsa_circ_0003159 and abated the effect of hsa_circ_0003159 on proliferation, migration, invasion and apoptosis in GC cells. Hsa_circ_0003159 promoted NDRG1 expression by competitively sponging miR-223-3p. Knockdown of NDRG1 reversed the suppressive effect of hsa_circ_0003159 on GC progression. Besides, hsa_circ_0003159 decreased GC cell xenograft tumor growth by regulating miR-223-3p and NDRG1.

**Conclusion:**

Hsa_circ_0003159 suppressed proliferation, migration, invasion and xenograft tumor growth but promoted apoptosis by decreasing miR-223-3p and increasing NDRG1 in GC, indicating a novel target for treatment of GC.

## Background

Gastric cancer (GC) is one of the most common cancers with a low 5-year survival rate (< 30%) [1, 2]. Recently, the diagnosis and treatment of GC have got significant progress [3, 4]. However, the prognosis of GC patients at advanced stages remains unsatisfactory. Therefore, exploring new targets for treatment of GC is excepted at the moment.

Circular RNAs (circRNAs) could act as essential targets for therapeutics of cancers [5]. CircRNAs are a class of noncoding RNAs, which are produced by the backsplicing of a link between upstream splice-acceptor site and downstream splice-donor site [6]. Moreover, circRNAs are widely and stably expressed and take part in the progression of GC [7]. For example, Huang et al. report that circRNA hsa_circ_0008035 could induce tumorigenesis of GC by promoting proliferation and invasion [8]. Furthermore, Liang et al. suggest that hsa_circ_006100 facilitates the tumor growth and metastasis of GC in vitro and in vivo [9]. The hsa_circ_0003159 is arose from CACNA2D1 gene located at chromosome 7:81689743–81746489, which is involved in the development of GC [10]. Nevertheless, the mechanism by which hsa_circ_0003159 mediating GC progression is largely unknown.

The competing endogenous RNA (ceRNA) hypothesis is an important mechanism allows circRNAs in cancers by sponging microRNAs (miRNAs) to modulate mRNAs [11]. Increasing evidences show that miR-223-3p could participate in regulating cell proliferation, migration and invasion in human cancers, including glioblastomas, renal cell carcinoma and colon cancer [12–14]. Importantly, miR-223-3p has been reported to promote the development of GC by promoting cell proliferation, migration and invasion [15, 16]. Moreover, N-myc downstream regulated gene 1 (NDRG1) is demonstrated as a metastasis suppressor and inhibit tumor malignancy of GC [17–19]. However, whether miR-223-3p and NDRG1 are associated with the mechanism of hsa_circ_0003159 is unknown. Interestingly, Circular RNA Interactome and starBase online predicted there are the same miR-223-3p binding sites between hsa_circ_0003159 and NDRG1. Hence, we hypothesized that the potential ceRNA network of hsa_circ_0003159/miR-223-3p/NDRG1 might be responsible for the mechanism of hsa_circ_0003159 in GC.

In this study, we detected the expression of hsa_circ_0003159 in GC tissues and cell lines and assessed the effect on proliferation, apoptosis, migration, invasion and xenograft tumor growth in GC. Furthermore, this work explored whether hsa_circ_0003159-mediated mechanism was associated with miR-223-3p and NDRG1.

## Materials and methods

### Patients and clinical samples

55 GC patients were recruited from the Fifth Affiliated Hospital of Sun Yat-Sen University and they all signed the written informed consents. The tumor tissues and adjacent normal samples were collected and stored at − 80 °C. The patients’ information was shown in Table 1. The overall survival and disease-free survival were analyzed after a 5-year follow-up. This research has received the approval of the Ethics Committee of the Fifth Affiliated Hospital of Sun Yat-Sen University.

**Table 1:** Statistics of hsa_circ_0003159 expression in gastric cancer tissues with clinicopathological factors of gastric cancer patients

Characteristics	n	hsa_circ_0003159		*P*
		High (n = 27)	Low (n = 28)	
Gender				
Male	30	12	18	0.140
Female	25	15	10	
Age (years)				
≥ 60	28	13	15	0.688
< 60	27	14	13	
Tumor size				
≥ 5 cm	29	11	17	0.139
< 5 cm	26	16	11	
Differentiation				
Well + moderate	20	13	7	0.074
Poor	35	14	21	
Lymph node metastasis				
Yes	21	6	15	0.017^*^
No	34	21	13	
Invasion depth				
T1 + T2	19	13	6	0.037^*^
T3 + T4	36	14	22	
TNM stage				
I + II	23	16	7	0.010^*^
III + IV	32	11	21	

### Cell culture

Human gastric mucosal epithelial cells (GES-1) and GC cell lines (NUGC-3, AGS, HS-746T and N87) were purchased from BeNa Culture Collection (Beijing, China). All cell lines were cultured in Roswell Park Memorial Institute (RPMI)-1640 (Thermo Fisher, Wilmington, DE, USA) containing 10% fetal bovine serum (Thermo Fisher) and 1% penicillin–streptomycin solution (Beyotime, Shanghai, China) at 37 °C in 5% CO_2_.

### Quantitative real-time polymerase chain reaction (qRT-PCR)

Total RNA was isolated by Trizol reagent (Solarbio, Beijing, China). The RNA separation of cytoplasmic and nuclear fractions was performed by Cytoplasmic & Nuclear RNA Purification Kit (Labomics, Nivelles, Belgium). For purity of circRNAs, the RNA was treated by RNase R (Geneseed, Guangzhou, China) for 20 min. 1 μg RNA was used for complementary DNA (cDNA) synthesis using the specific TaqMan cDNA synthesis kit (Thermo Fisher). The cDNA products were diluted by 1:5 and then mixed with SYBR (Thermo Fisher) and specific primers (Sangon, Shanghai, China) for qRT-PCR. 18S rRNA and U6 were regarded as endogenous controls. The primers were listed as: hsa_circ_0003159: Forward, 5′-CCGAACATCTGTCTCCGAAA-3′; Reverse, 5′-CTGCTGCGTGCTGATAAGAT-3′; CACNA2D1: Forward, 5′-CAGTTGAGATGGAGGATGATG-3′; Reverse, 5′-TTGTATGAGCAGTCGTGTGTC-3′; NDRG1: Forward, 5′-ACACCTACCGCCAGCACATT-3′; Reverse, 5′-GGTCGCTCAATCTCCAGGTC-3′; miR-223-3p: Forward, 5′-AGCTGGTGTTGTGAATCAGGCCG-3′; Reverse, 5′-TGGTGTCGTGGAGTCG-3′; 18S rRNA (Forward, 5′-GGCCCTGTAATTGGAATGAGTC-3′; Reverse, 5′-CCAAGATCCAACTACGAGCTT-3′); U6 (Forward, 5′-CTCGCTTCGGCAGCACATATACT-3′; Reverse, 5′-ACGCTTCACGAATTTGCGTGTC-3′). The relative RNA levels were calculated by 2^−ΔΔCt^ method [20].

### Cell transfection

The overexpression vector of hsa_circ_0003159 was constructed using pLCDH-ciR vector (Geneseed), with the empty vector (vector) as negative control. The short interfering RNA (siRNA) for hsa_circ_0003159 (si-hsa_circ_0003159, 5′-AGCUUCACCUGAGAAAAAUGA-3′), siRNA for NDRG1 (si-NDRG1, 5′-GCUGAAGCUCGUCAGUUCACCAUCC-3′), siRNA negative control (si-NC, 5′-UUCUCCGAACGUGUCACGU-3′), miR-223-3p mimic (miR-223-3p, 5′-UGUCAGUUUGUCAAAUACCCCA-3′), mimic negative control (miR-NC, 5′-GUGGAUUUUCCUCUAUGAUUU-3′), miR-223-3p inhibitor (anti-miR-223-3p, 5′-UGGGGUAUUUGACAAACUGACA-3′), and inhibitor negative control (anti-NC, 5′-CAGUACUUUUGUGUAGUACAA-3′) were synthesized by GenePharma (Shanghai, China). These vectors or oligonucleotides were transfected into NUGC-3 and AGS cells using Lipofectamine™ 2000 transfection reagent (Thermo Fisher) for 24 h.

### Cell counting kit (CCK)-8

NUGC-3 and AGS cells (1 × 10^4^ cells per well) were seeded into 96-well plates and then cultured for 24, 48 and 72 h. Next, 10 μL of CCK-8 solution (MedChemExpress, Monmouth, NJ, USA) was added to each well and cells were cultured for another 2 h. The optical density value at 450 nm was detected by a microplate reader (Potenov, Beijing, China).

### Flow cytometry

NUGC-3 and AGS cells (5 × 10^4^ cells per well) in 12-well plates were incubated at 37 °C for 72 h. Subsequently, cells were collected and resuspended in Annexin V-fluorescein isothiocyanate (FITC) binding buffer, followed by incubation of 5 μL of Annexin V-FITC and propidium iodide (PI) (Beyotime) for 10 min. The apoptotic cells at upper and lower right quadrants were examined with a flow cytometer (Countstar, Shanghai, China). For cell cycle analysis, NUGC-3 and AGS cells (5 × 10^5^ cells per well) were cultured into 6-well plates for 72 h. Then cells were fixed with 70% ethanol and incubated with 50 μg/mL PI and RNase A for 20 min. Next, cell cycle distribution was detected via flow cytometer.

### Transwell assay

The abilities of migration and invasion were determined by transwell chambers (Corning, Corning, NY, USA). For migration assay, NUGC-3 and AGS cells (2 × 10^5^ cells/mL) were resuspended in serum-free RPMI-1640 medium and 100 μL of cell suspension was plated onto the upper chambers, while 500 μL of medium containing 10% fetal bovine serum was added to the lower chambers. After incubation for 24 h, cells migrated the membranes were fixed and stained with 0.1% crystal violet (Sigma, St. Louis, MO, USA). Three random fields were selected under the microscope (Olympus, Tokyo, Japan) for counting the number of stained cells. For invasion assay, transwell chambers were precoated with Matrigel, and the procedures were similar with migration assay.

### Western blot

The pre-cold radio immunoprecipitation assay lysis buffer (Beyotime) containing protease inhibitor was used to isolate the total proteins. After the quantification and denaturation, 20 μg protein samples were separated by sodium dodecyl sulfate–polyacrylamide gel electrophoresis, and transfected onto polyvinylidene difluoride membranes (Millipore, Billerica, MA, USA). The membranes were interacted with 5% skim milk for blocking the non-specific sites, and then incubated with primary antibodies at 4 °C and corresponding secondary antibody. The antibodies were purchased from Abcam (Cambridge, UK) as follows: anti-CyclinD1 (ab226977, 1:2000 dilution), anti-Cleaved-caspase-3 (ab2302, 1:1000 dilution), anti-matrix metalloproteinase-9 (MMP-9) (ab73734, 1:1000 dilution), anti-NDRG1 (ab196621, 1:2000 dilution), anti-GAPDH (ab37168, 1:2000 dilution) and horseradish peroxidase-labeled IgG (ab205718, 1:10000 dilution). GAPDH was used as an internal control. The blots were visualized by enhanced chemiluminescence reagent (Beyotime) and analyzed by QuantityOne software (Bio-Rad, Hercules, CA, USA).

### Dual-luciferase reporter assay

Circular RNA Interactome and starBase were used to predict the potential targets of hsa_circ_0003159 and miR-223-3p, respectively. The hsa_circ_0003159 sequences and NDRG1 3′ untranslated region (UTR) containing miR-223-3p binding sites were cloned into the downstream of psiCHECK-2 luciferase reporter vector (Promega, Madison, WI, USA) to form the wild-type (WT) luciferase reporter vectors WT hsa_circ_0003159 and NDRG1 3′UTR-WT, respectively. The mutant (MUT) luciferase reporter constructs MUT hsa_circ_0003159 and NDRG1 3′UTR-MUT were generated by mutating the binding sites of miR-223-3p. For dual-luciferase reporter assay, NUGC-3 and AGS cells were co-transfected with these constructs and miR-223-3p or miR-NC. After 48 h, luciferase activity was measured by a dual-luciferase assay system (Promega).

### Xenograft model

The animal research was conducted under the experimental animal use guidelines and has gained the approval of the ethics committee of the Fifth Affiliated Hospital of Sun Yat-Sen University. Five-week-old male BALB/c nude mice (Charles River, Beijing, China) were used for xenograft model establishment. The lentiviral vector of hsa_circ_0003159 was constructed using pLCDH-ciR vector (Geneseed), and the empty vector (vector) was used as negative control. NUGC-3 cells were infected with hsa_circ_0003159 or vector, and the stably transfected cells were selected by puromycin. NUGC-3 cells (2 × 10^6^ cells per mouse) stably transfected with hsa_circ_0003159 or vector were subcutaneously injected into the mice (n = 5 per group). Tumor grew 28 days and the volume was measured every 7 days, which was calculated with a formula: volume = length × width^2^/2. At the ending point, all mice were killed and tumor tissues were weighed. Moreover, the collected tissues were used for hematoxylin–eosin staining or analyses of qRT-PCR and western blot.

### Statistical analysis

The experiment was executed more than 3 times. All data were shown as mean ± standard deviation (S.D.). GraphPad Prism 7 software (GraphPad Inc., La Jolla, CA, USA) was used for statistical analysis. The overall survival and disease-free survival of GC patients were analyzed via Kaplan–Meier plot and log-rank test. The association between hsa_circ_0003159 expression and clinicopathological factors was analyzed via χ2 test. The independent prognostic factors were analyzed via multivariate analysis. The linear association among the levels of hsa_circ_0003159-miR-223-3p, NDRG1-miR-223-3p and NDRG1-hsa_circ_0003159 was analyzed by Spearman’s correlation coefficient. Student’s *t* test and one-way analysis of variance were used for the comparison between groups. The difference was considered significant when *P *< 0.05.

## Results

### Hsa_circ_0003159 expression is reduced in GC

**Fig. 1:**
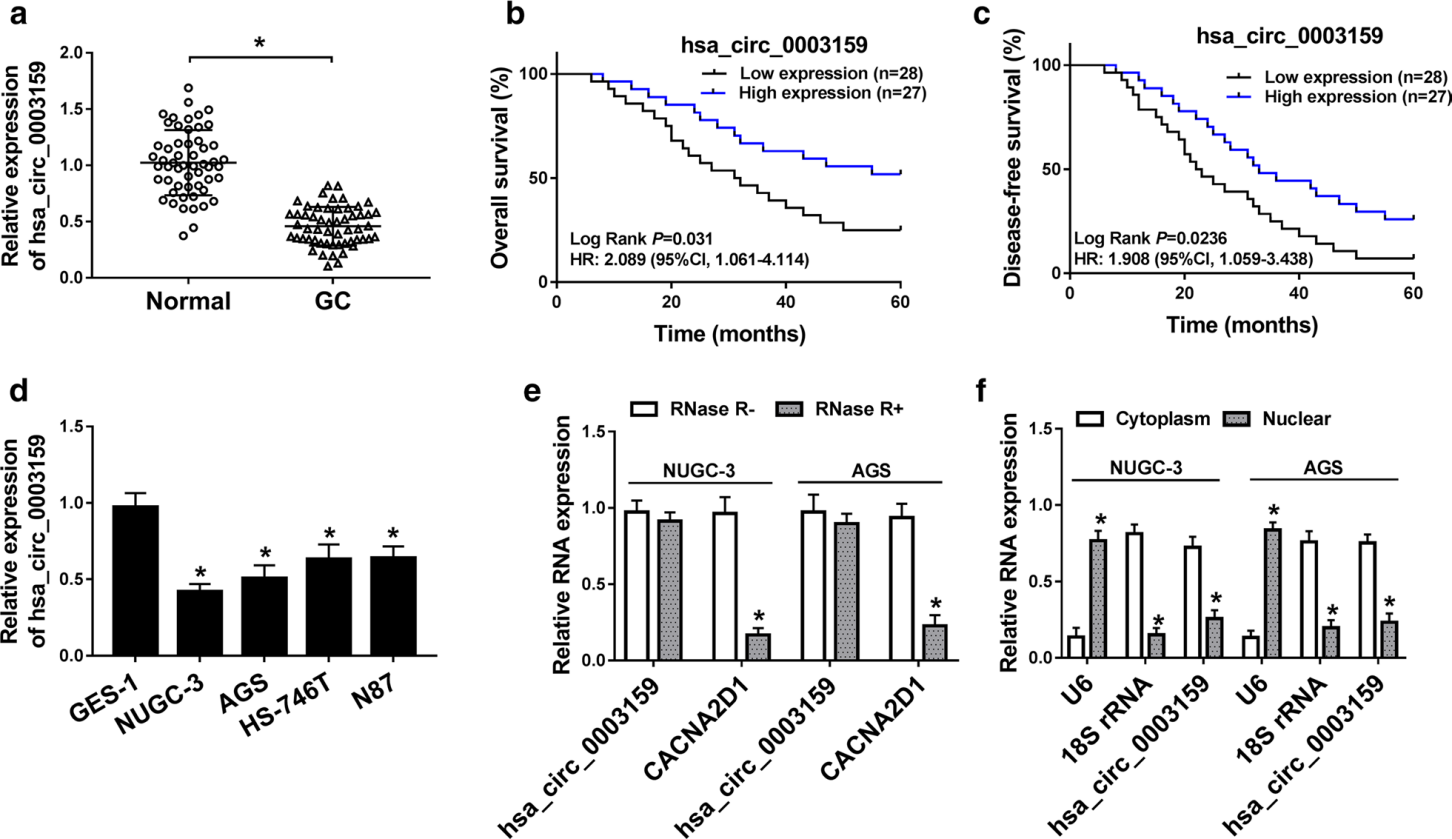
The expression of hsa_circ_0003159 is decreased in GC. **a** The expression of hsa_circ_0003159 was detected in GC tissues and normal tissues (n = 55) by qRT-PCR. **b**, **c** Overall survival (**b**) and disease-free survival (**c**) of patients were analyzed according to the expression of hsa_circ_0003159. *HR* hazards ratio, *CI* confidence interval. **d** The level of hsa_circ_0003159 was detected in GC cell lines (NUGC-3, AGS, HS-746T and N87) and normal GES-1 cells by qRT-PCR. **e** The expression levels of hsa_circ_0003159 and CACNA2D1 were detected in NUGC-3 and AGS cells after treatment of RNase R or not by qRT-PCR. **f** The abundance of hsa_circ_0003159 was examined in NUGC-3 and AGS cells by qRT-PCR, with U6 and 18S rRNA as internal controls for nuclear and cytoplasm, respectively. *P < 0.05

To measure the expression of hsa_circ_0003159 in GC, 55 paired GC tissues and normal samples were collected. The data of qRT-PCR showed that the abundance of hsa_circ_0003159 was significantly decreased in GC tissues when compared to that in normal samples (Fig. 1a). Moreover, the GC patients were divided into high expression group (n = 27) and low expression group (n = 28) according to the median of hsa_circ_0003159 level. The low expression group displayed lower overall survival (*P *= 0.031; HR = 2.089 (95% CI 1.061–4.114)) and disease-free survival (*P *= 0.0236; HR = 1.908 (95% CI 1.059–3.438)) than high expression group after a 5-year follow-up (Fig. 1b, c). Meanwhile, low expression of hsa_circ_0003159 was associated with lymph node metastasis, invasion depth and tumor-node-metastasis (TNM) stage (Table 1). Multivariate analysis revealed that lymph node metastasis, invasion depth, TNM stage and low hsa_circ_0003159 were independent risk factors for overall survival of patients (Table 2). In addition, 4 GC cell lines (NUGC-3, AGS, HS-746T and N87) displayed lower level of hsa_circ_0003159 than control GES-1 cells (Fig. 1d). The NUGC-3 and AGS cells with relative lower expression of hsa_circ_0003159 were used for further experiments. Furthermore, hsa_circ_0003159 showed more resistant to RNase R than corresponding linear-CACNA2D1, revealed by the sharp reduction of linear form and few changes of circular form in NUGC-3 and AGS cells (Fig. 1e). Besides, by detecting the abundance of hsa_circ_0003159 in cytoplasm and nuclear, the results showed that this circRNA was mainly localized in cytoplasm (Fig. 1f).

**Table 2:** Multivariate analysis of risk factors for survival of patients with gastric cancer

Characteristics	Multivariate analysis	
	HR (95% CI)	*P*
Lymph node metastasis	1.85 (1.13–3.47)	0.029
Invasion depth	2.47 (1.19–6.39)	0.016
TNM stage	2.59 (1.21–6.51)	0.018
Hsa_circ_0003159	0.46 (0.32–0.79)	0.011

*CI* confidence interval, *HR* hazard ratio, *TNM* tumor-node-metastasis

### Hsa_circ_0003159 suppresses proliferation, migration and invasion but promotes apoptosis in GC cells

**Fig. 2:**
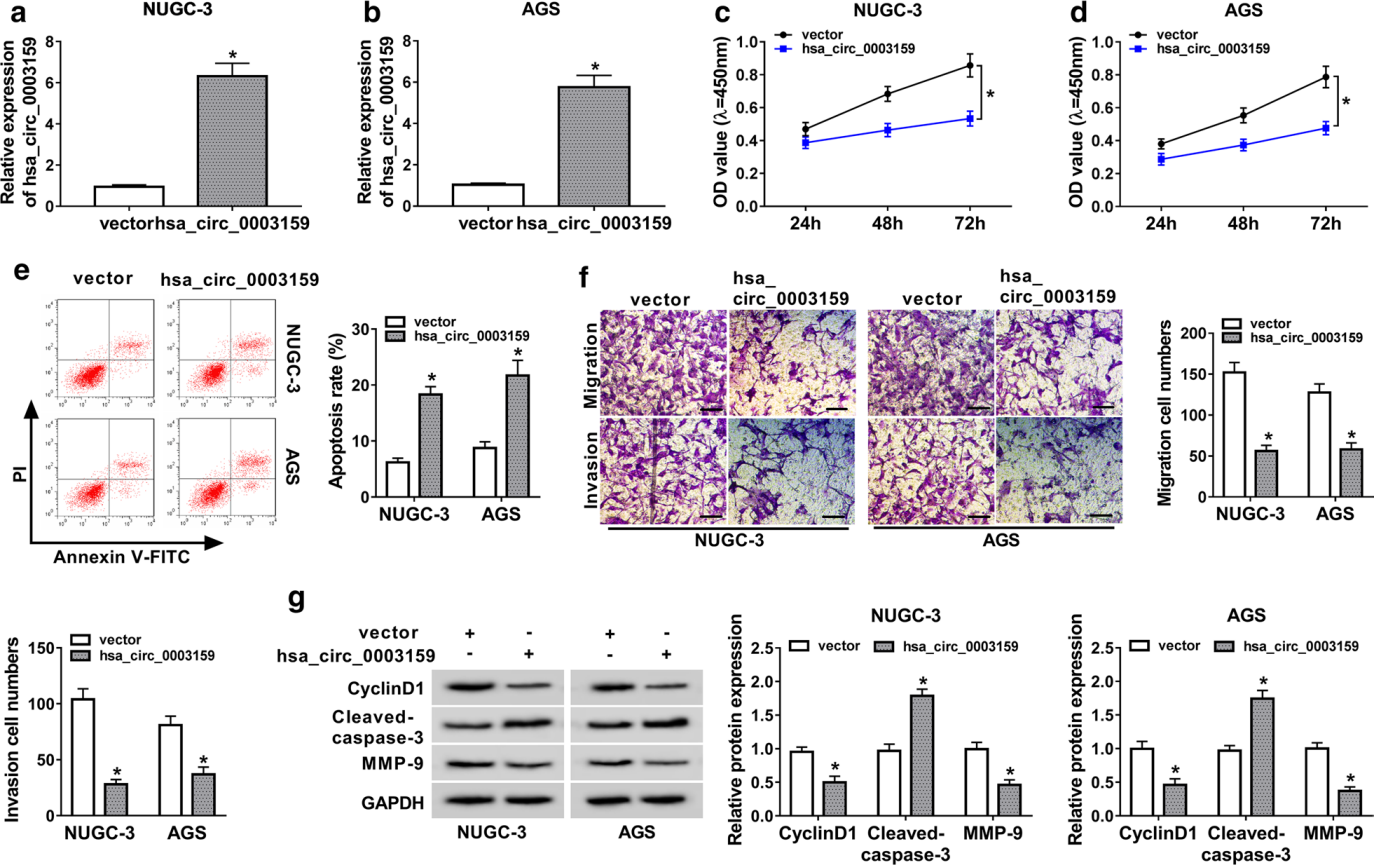
Overexpression of hsa_circ_0003159 inhibits proliferation, migration and invasion but induces apoptosis in GC cells. **a**, **b** The expression of hsa_circ_0003159 was detected in NUGC-3 and AGS cells transfected with hsa_circ_0003159 or vector by qRT-PCR. **c**, **d** The proliferation of NUGC-3 and AGS cells transfected with hsa_circ_0003159 or vector was measured at 24, 48 and 72 h by CCK-8 assay. **e** The apoptotic rate of NUGC-3 and AGS cells transfected with hsa_circ_0003159 or vector was detected at 72 h by flow cytometry. **f** The migrated and invasive abilities of NUGC-3 and AGS cells transfected with hsa_circ_0003159 or vector were analyzed at 24 h by transwell assay. Scale bar: 50 μm. **g** The protein levels of CyclinD1, Cleaved-caspase-3 and MMP-9 were measured in NUGC-3 and AGS cells transfected with hsa_circ_0003159 or vector at 72 h by western blot. *P < 0.05

To explore the role of hsa_circ_0003159 in GC, this circRNA was overexpressed in NUGC-3 and AGS cells by transfection of hsa_circ_0003159 overexpression vector. The transfection efficacy was confirmed by about sixfold increase of hsa_circ_0003159 level in hsa_circ_0003159 group compare with vector group (Fig. 2a, b). Furthermore, the data of CCK-8 assay displayed that overexpression of hsa_circ_0003159 significantly decreased the proliferation of NUGC-3 and AGS cells at 72 h (Fig. 2c, d). In addition, up-regulation of hsa_circ_0003159 remarkably induced apoptosis production in NUGC-3 and AGS cells at 72 h (Fig. 2e). In addition, overexpression of hsa_circ_0003159 induced cell cycle arrest at G1 phase (Additional file 1: Figure S1). Moreover, the migrated and invasive abilities of NUGC-3 and AGS cells were obviously inhibited by addition of hsa_circ_0003159 at 24 h (Fig. 2f). Meanwhile, hsa_circ_0003159 evidently repressed epithelial-mesenchymal transition (Additional file 2: Figure S2). Besides, the protein levels of pro-proliferation CyclinD1, pro-apoptosis Cleaved-caspase-3 and pro-migration MMP-9 were detected in NUGC-3 and AGS cells. Results showed that overexpression of hsa_circ_0003159 led to significant reduction of CyclinD1 and MMP-9 and increase of Cleaved-caspase-3 in the two cell lines (Fig. 2g).

### Hsa_circ_0003159 acts as a sponge of miR-223-3p in GC cells

**Fig. 3:**
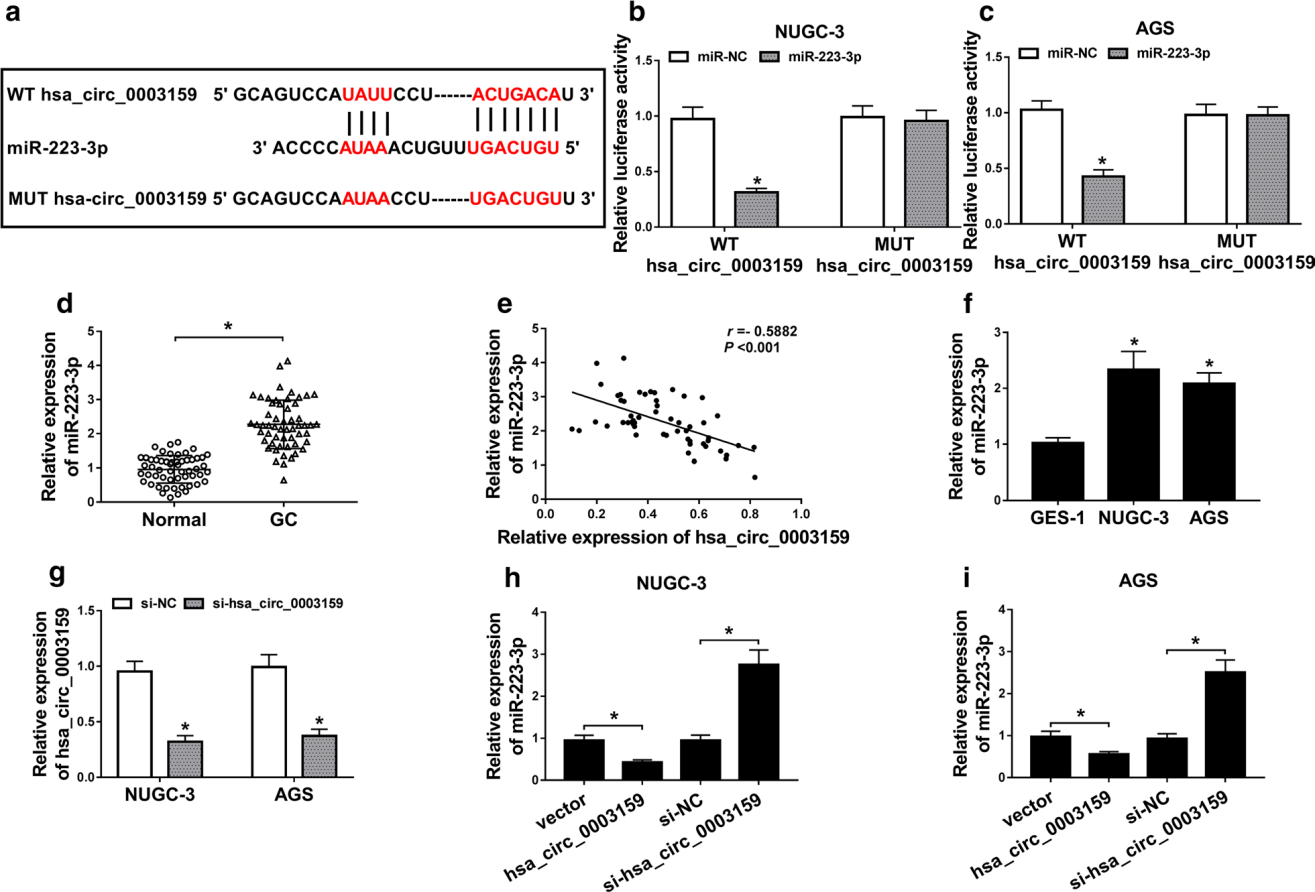
Hsa_circ_0003159 is a sponge of miR-223-3p in GC cells. **a** The binding sites of hsa_circ_0003159 and miR-223-3p were predicted by Circular RNA Interactome. **b**, **c** Luciferase activity was measured in NUGC-3 and AGS cells co-transfected with WT hsa_circ_0003159 or MUT hsa_circ_0003159 and miR-NC or miR-223-3p. **d** The expression of miR-223-3p was detected in GC tissues and normal samples (n = 55) by qRT-PCR. **e** The linear association between the levels of hsa_circ_0003159 and miR-223-3p in GC tissues was analyzed. **f** The level of miR-223-3p was examined in GC cell lines (NUGC-3 and AGS) and GES-1 cells by qRT-PCR. **g** The abundance of hsa_circ_0003159 was measured in NUGC-3 and AGS cells transfected with si-NC or si-hsa_circ_0003159 by qRT-PCR. **h**, **i** The expression of miR-223-3p was detected in NUGC-3 and AGS cells transfected with vector, hsa_circ_0003159, si-NC or si-hsa_circ_0003159 by qRT-PCR. *P < 0.05

Seeing that hsa_circ_0003159 was mainly located in cytoplasm, the potential miRNAs sponged by hsa_circ_0003159 were explored by Circular RNA Interactome. It was predicted that miR-223-3p had the binding sites of hsa_circ_0003159 (Fig. 3a). To validate the association between hsa_circ_0003159 and miR-223-3p, the luciferase reporter vectors WT hsa_circ_0003159 and MUT hsa_circ_0003159 were generated and dual-luciferase reporter assay was performed in NUGC-3 and AGS cells. As shown in Fig. 3b, c, the luciferase activity was declined more than 60% by miR-223-3p overexpression in WT hsa_circ_0003159 group in the two cell lines, while it showed little effect in MUT hsa_circ_0003159 group. Moreover, the expression of miR-223-3p was markedly enhanced in GC tissues compared with that in normal group (Fig. 3d). Meanwhile, there was an inverse correlation between the levels of hsa_circ_0003159 and miR-223-3p in GC tissues (r = − 0.5882, *P *< 0.001) (Fig. 3e). NUGC-3 and AGS cells also displayed high expression of miR-223-3p than GES-1 cells (Fig. 3f). In order to investigate the effect of hsa_circ_0003159 on miR-223-3p expression, NUGC-3 and AGS cells were transfected with vector, hsa_circ_0003159, si-NC or si-hsa_circ_0003159. The knockdown efficacy of hsa_circ_0003159 was validated in Fig. 3g. Additionally, miR-223-3p expression in NUGC-3 and AGS cells was evidently decreased by hsa_circ_0003159 overexpression and increased by hsa_circ_0003159 knockdown (Fig. 3h, i).

### Overexpression of miR-223-3p attenuates the effect of hsa_circ_0003159 on proliferation, apoptosis, migration and invasion in GC cells

**Fig. 4:**
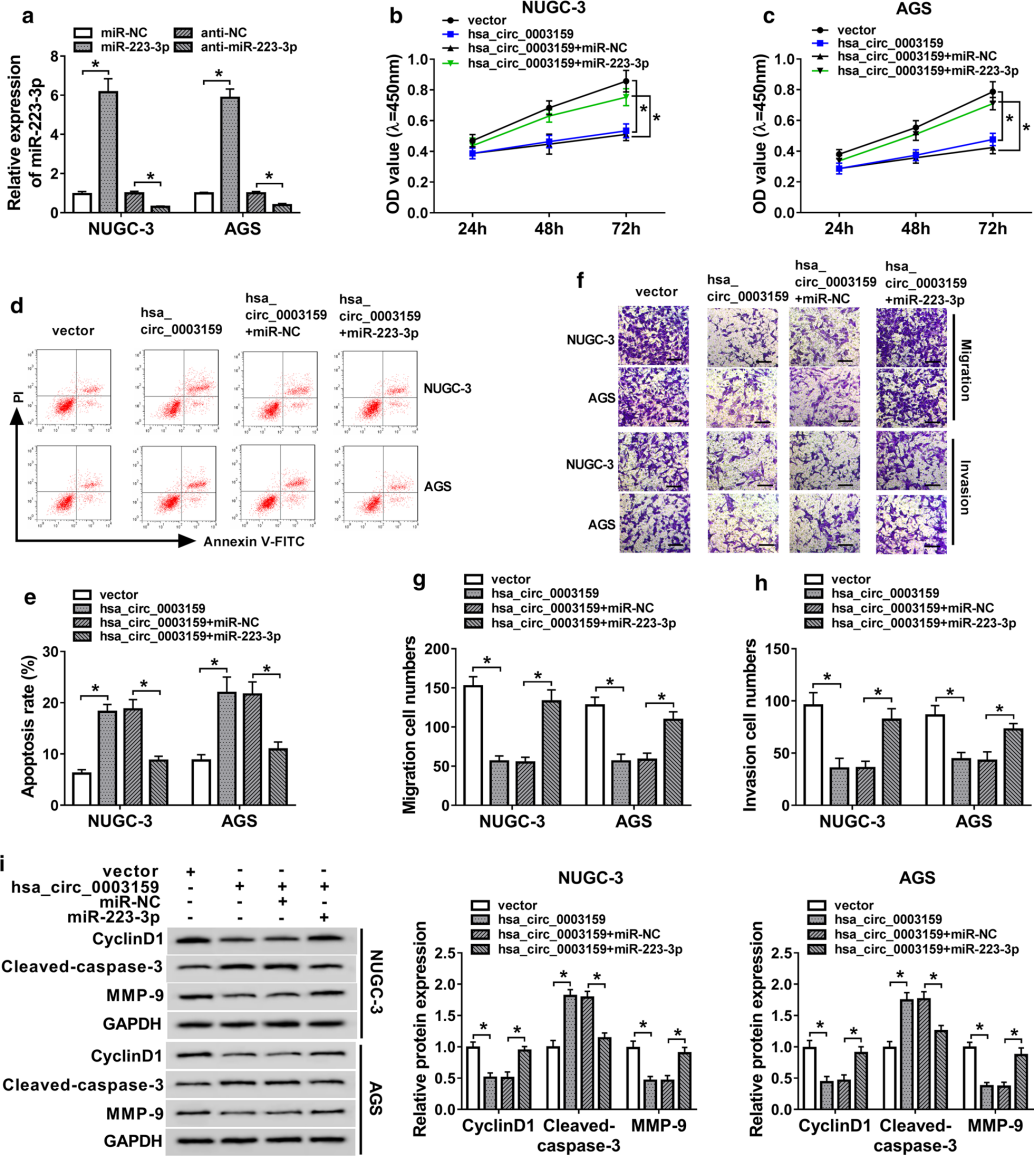
Up-regulation of miR-223-3p reverses the effect of hsa_circ_0003159 on proliferation, migration, invasion and apoptosis in GC cells. **a** The expression of miR-223-3p was measured in NUGC-3 and AGS cells transfected with miR-NC, miR-223-3p, anti-NC or anti-miR-223-3p by qRT-PCR. **b**–**g** Cell proliferation (**b**, **c**), apoptosis (**d**, **e**), migration and invasion (**f**–**h**) and protein levels of CyclinD1, Cleaved-caspase-3 and MMP-9 (**i**) were detected in NUGC-3 and AGS cells transfected with vector, hsa_circ_0003159, hsa_circ_0003159 and miR-NC or miR-223-3p by CCK-8, flow cytometry, transwell and western blot assays, respectively. *P < 0.05

The transfection efficacy of miR-223-3p and anti-miR-223-3p was confirmed in Fig. 4a. To explore whether miR-223-3p was required for hsa_circ_0003159-meidated mechanism, NUGC-3 and AGS cells were transfected with vector, hsa_circ_0003159, hsa_circ_0003159 and miR-NC or miR-223-3p mimic. As shown in Fig. 4b, c, the up-regulation of miR-223-3p abated the suppressive role of hsa_circ_0003159 in proliferation of NUGC-3 and AGS cells. Moreover, miR-223-3p addition mitigated cell apoptosis induced by hsa_circ_0003159 overexpression in the two cell lines (Fig. 4d, e). Additionally, the impaired abilities of migration and invasion induced by hsa_circ_0003159 was restored by miR-223-3p overexpression in NUGC-3 and AGS cells (Fig. 4f–h). Besides, the regulatory effect of hsa_circ_0003159 on CyclinD1, Cleaved-caspase-3 and MMP-9 protein levels was weakened by miR-223-3p overexpression (Fig. 4i).

### Hsa_circ_0003159 promotes NDRG1 expression by regulating miR-223-3p in GC cells

**Fig. 5:**
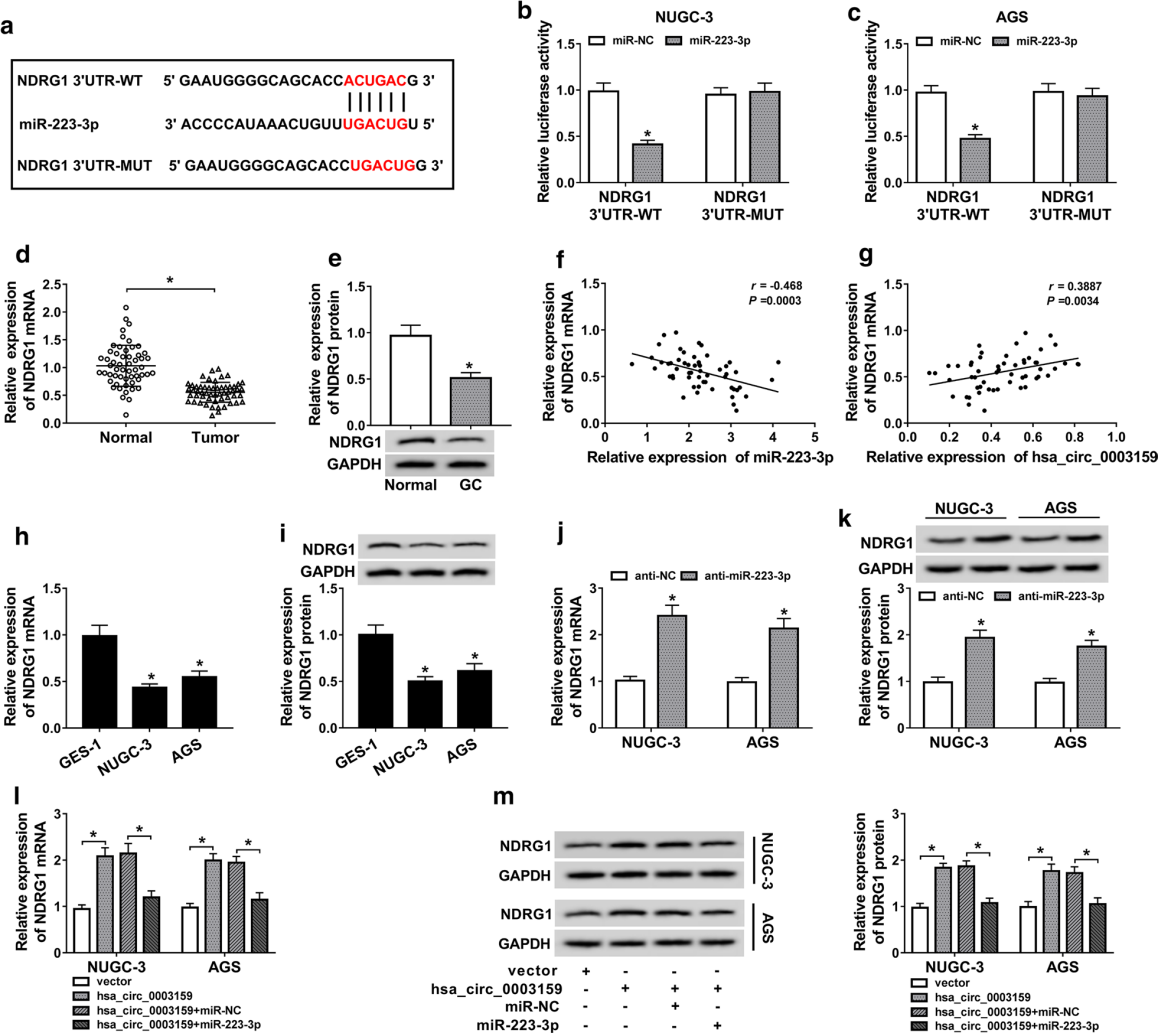
NDRG1 is regulated by hsa_circ_0003159 and miR-223-3p in GC cells. **a** The binding sites of miR-223-3p and NDRG1 were predicted by starBase. **b**, **c** Luciferase activity was measured in NUGC-3 and AGS cells co-transfected with NDRG1 3′UTR-WT or NDRG1 3′UTR-MUT and miR-NC or miR-223-3p. **d**, **e** The expression levels of NDRG1 mRNA and protein were detected in GC tissues and normal tissues by qRT-PCR and western blot. **f**, **g** The association between expression of NDRG1 mRNA and miR-223-3p or hsa_circ_0003159 in GC tissues was assessed. **h**, **i** The mRNA and protein levels of NDRG1 were measured in GC cells (NUGC-3 and AGS) and GES-1 cells by qRT-PCR and western blot. **j**, **k** The mRNA and protein levels of NDRG1 were measured in NUGC-3 and AGS cells transfected with anti-miR-223-3p or anti-NC by qRT-PCR and western blot. **l**, **m** The mRNA and protein levels of NDRG1 were examined in NUGC-3 and AGS cells transfected with vector, hsa_circ_0003159, hsa_circ_0003159 and miR-NC or miR-223-3p by qRT-PCR and western blot. *P < 0.05

To further elucidate the ceRNA network mediated by hsa_circ_0003159, the target s of miR-223-3p were explored by starBase, which showed that NDRG1 had the miR-223-3p binding sites (Fig. 5a). For validation of the target association between miR-223-3p and NDRG1, the luciferase reporter vectors NDRG1 3′UTR-WT and NDRG1 3′UTR-MUT were constructed and transfected into NUGC-3 and AGS cells. The analysis of dual-luciferase reporter described that miR-223-3p overexpression led to great loss of luciferase activity in NDRG1 3′UTR-WT group, whereas it did not affect the activity in NDRG1 3′UTR-MUT group (Fig. 5b, c). Moreover, the mRNA and protein levels of NDRG1 were significantly reduced in GC tissues compared with those in normal samples (Fig. 5d, e). Meanwhile, the mRNA level of NDRG1 was negatively associated with miR-223-3p level (r = − 0.468, *P *= 0.0003) and positively corelated with hsa_circ_0003159 expression (r = 0.3887, *P *= 0.0034) in GC tissues (Fig. 5f, g). In addition, the expression of NDRG1 was also evidently decreased at transcriptional and protein levels in NUGC-3 and AGS cells than that in GES-1 cells (Fig. 5h, i). Furthermore, the abundances of NDRG1 mRNA and protein in NUGC-3 and AGS cells were significantly elevated by miR-223-3p knockdown (Fig. 5j, k). Besides, the mRNA and protein levels of NDRG1 were greatly enhanced by hsa_circ_0003159 overexpression, which was weakened by introduction of miR-223-3p in NUGC-3 and AGS cells (Fig. 5l, m).

### Silence of NDRG1 alleviates the effect of has_circ_0003159 on proliferation, migration, invasion and apoptosis in GC cells

**Fig. 6:**
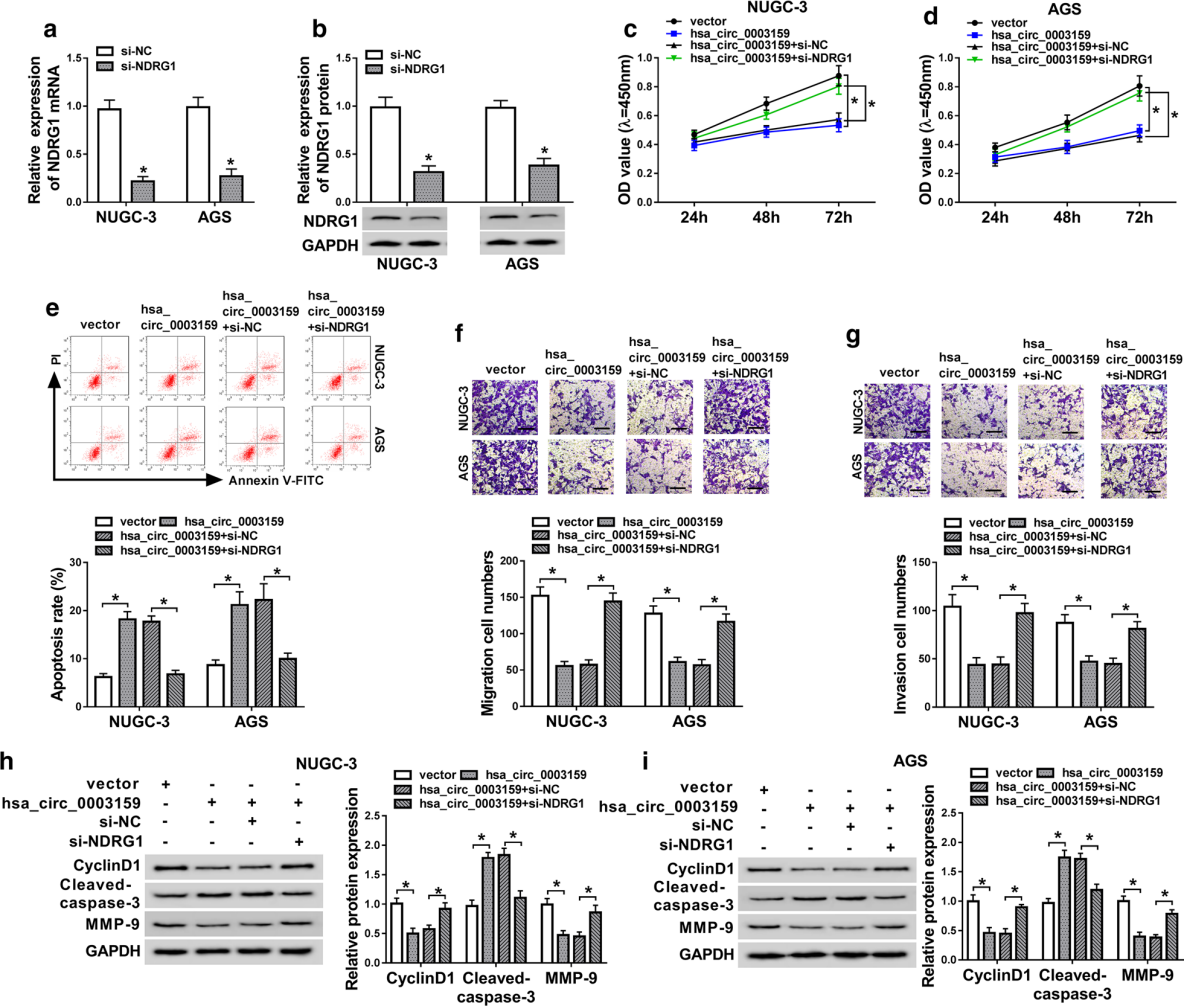
Knockdown of NDRG1 attenuates the effect of hsa_circ_0003159 on proliferation, migration and apoptosis in GC cells. **a**, **b** The mRNA and protein levels of NDRG1 were measured in NUGC-3 and AGS cells transfected with si-NC or si-NDRG1 by qRT-PCR and western blot. Cell proliferation (**c**, **d**), apoptosis (**e**), migration (**f**), invasion (**g**) and protein levels of CyclinD1, Cleaved-caspase-3 and MMP-9 (**h**, **i**) were examined in NUGC-3 and AGS cells transfected with vector, hsa_circ_0003159, hsa_circ_0003159 and si-NC or si-NDRG1 by CCK-8, flow cytometry, transwell and western blot assays, respectively. *P < 0.05

To explore whether hsa_circ_0003159-regulated GC progression was mediated by NDRG1, NUGC-3 and AGS cells were transfected with vector, hsa_circ_0003159, hsa_circ_0003159 and si-NC or si-NDRG1. The suppressive efficacy of si-NDRG1 on NDRG1 expression in NUGC-3 and AGS cells was confirmed by sharply decreased NDRG1 in si-NDRG1 group (Fig. 6a, b). Furthermore, silence of NDRG1 abated the suppressive effect of hsa_circ_0003159 on proliferation of NUGC-3 and AGS cells (Fig. 6c, d). In addition, hsa_circ_0003159-induced apoptosis was weakened by down-regulation of NDRG1 in NUGC-3 and AGS cells (Fig. 6e). Moreover, interference of NDRG1 abrogated the inhibitive effect of hsa_circ_0003159 on migration and invasion of NUGC-3 and AGS cells (Fig. 6f, g). Besides, the reduction of CyclinD1 and MMP-9 and increase of Cleaved-caspase-3 mediated by hsa_circ_0003159 were reversed by NDRG1 silence in the two cell lines (Fig. 6h, i).

### Hsa_circ_0003159 reduces GC cell xenograft tumor growth

**Fig. 7:**
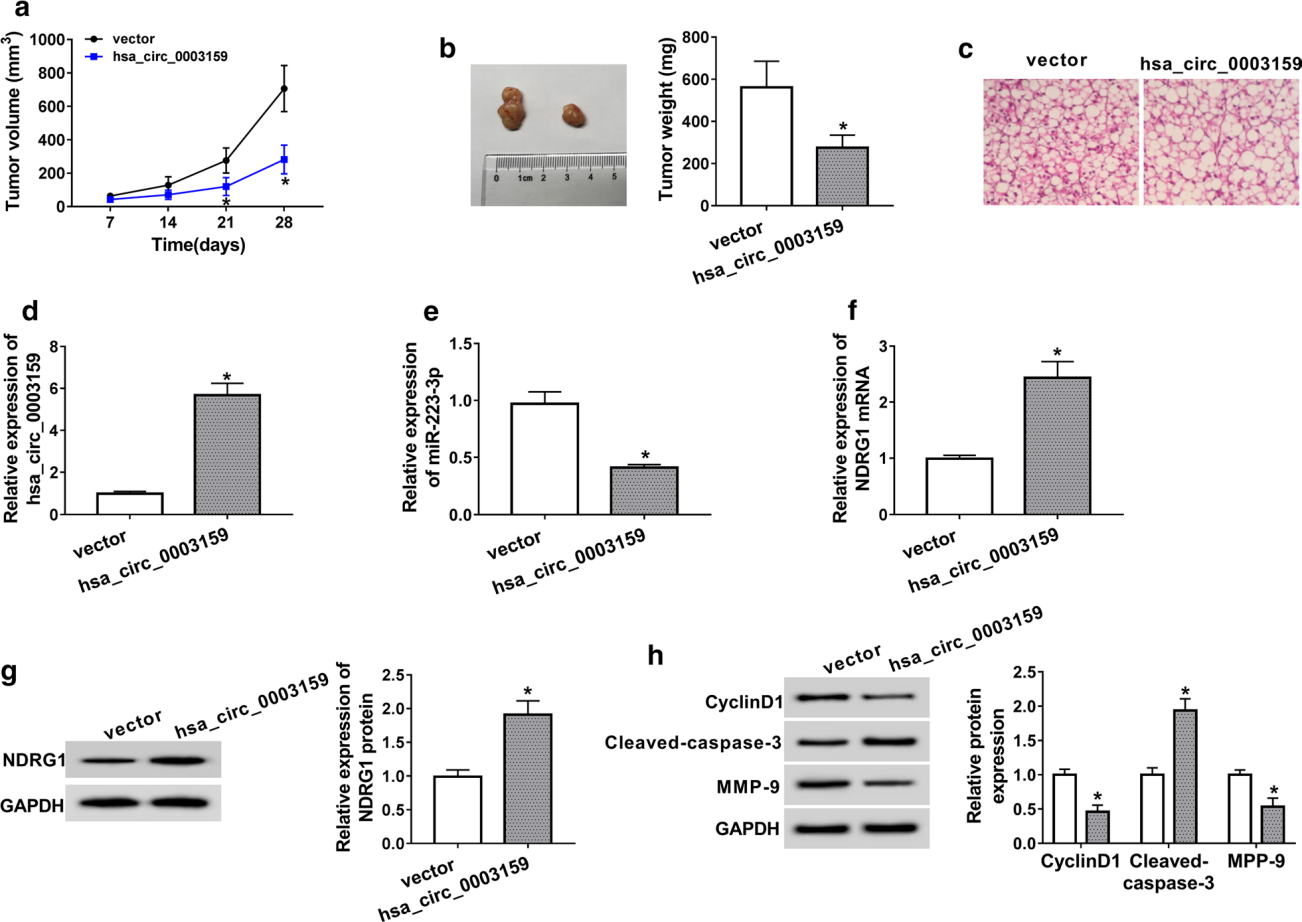
Hsa_circ_0003159 decreases GC cell xenograft tumor growth. **a** Tumor volume was measured every week. **b** Tumor weight was detected in each group at the ending point. **c** The representative images of hematoxylin–eosin staining. **d**–**g** The expression levels of hsa_circ_0003159, miR-223-3p, NDRG1 mRNA and protein were examined in each group by qRT-PCR and western blot. **h** The protein levels of CyclinD1, Cleaved-caspase-3 and MMP-9 were detected in each group by western blot. *P < 0.05

To further investigate the effect of hsa_circ_0003159 on GC progression in vivo, NUGC-3 cells stably transfected with vector or hsa_circ_0003159 were subcutaneously injected into nude mice, named as vector or hsa_circ_0003159 group, respectively. The tumor volume was significantly decreased at 21 and 28 days in hsa_circ_0003159 group compared with that in vector group (Fig. 7a). Moreover, at the ending point, tumor weight of hsa_circ_0003159 group was lower than that in vector group (Fig. 7b). The hematoxylin–eosin staining showed that these were less malignant cells in hsa_circ_0003159 group than vector group (Fig. 7c). Additionally, the expression levels of hsa_circ_0003159, miR-223-3p and NDRG1 were detected in tumor tissues of each group. The results showed that the levels of hsa_circ_0003159 and NDRG1 were significantly increased but miR-223-3p expression was decreased in hsa_circ_0003159 group compared with those in vector group (Fig. 7d–g). Furthermore, down-regulation of CyclinD1 and MMP-9 and elevation of Cleaved-caspase-3 were shown in hsa_circ_0003159 group compared with that in vector group (Fig. 7h).

## Discussion

GC is a public problem with high incidence and mortality [21]. CircRNAs have the critical clinical significance in GC [22]. The former work suggested that down-regulation of hsa_circ_0003159 had the potential clinical value in GC [10]. However, how and whether hsa_circ_0003159 mediated GC progression remain unclear. Here we found that hsa_circ_0003159 was lowly expressed and associated with poor outcome of patients in GC. CyclinD1 was a proliferation-associated marker of GC cells by promoting cell cycle process [23]. Caspase-3 activation mediated cell apoptosis in many cell lines in response to some stimuli [24]. MMP-9 activity was associated with the abilities of migration and invasion of GC cells [25]. By detecting their levels and performing the functional assays, we found that hsa_circ_0003159 inhibited cell proliferation, migration and invasion but promoting apoptosis of GC cells, disclosing that hsa_circ_0003159 could be used as a tumor suppressor in GC. However, the underlying mechanism of hsa_circ_0003159 remains undetermined. In the current work, we were the first to confirm the ceRNA network of hsa_circ_0003159/miR-223-3p/NDRG1 in GC cells.

In this study, we found that hsa_circ_0003159 was mainly expressed in cytoplasm of GC cells, indicating that hsa_circ_0003159 could function as a sponge of miRNAs. Previous studies demonstrated that miR-223-3p played essential role in human cancers by promoting or inhibiting cancer development [12–14]. Here we first confirmed that hsa_circ_0003159 could sponged and negatively regulated miR-223-3p in GC cells. Moreover, we found that miR-223-3p expression was increased in GC, indicating the high expression of miR-223-3p might contribute to the development of GC, which was also in agreement with former works that displayed the pro-proliferation and pro-metastasis role of miR-223-3p in GC cells [15, 26]. Similarly, we also found that up-regulation of miR-223-3p reversed the suppressive effect of hsa_circ_0003159 by its pro-cancer function. Meanwhile, these findings suggested that hsa_circ_0003159 repressed GC progression by sponging miR-223-3p in vitro.

Next, we further explored the ceRNA network mediated by hsa_circ_0003159 in GC cells. Having given that hsa_circ_0003159 could sponge miR-223-3p, we explored the targets of miR-223-3p and confirmed that NDRG1 as a target of miR-223-3p had the same binding sites with hsa_circ_0003159. In addition, we validated that hsa_circ_0003159 could promote NDRG1 expression by competitively binding to miR-223-3p, supporting the ceRNA of hsa_circ_0003159/miR-223-3p/NDRG1 in GC cells. NDRG1 has been regarded as a metastasis suppressor in human cancers [17]. Besides, the rescue experiments displayed that NDRG1 knockdown attenuated the suppressive effect of hsa_circ_0003159 on GC progression, indicating the inhibitive role of NDRG1 in GC, which was also in agreement with previous studies [18, 27]. This also uncovered that hsa_circ_0003159 regulated GC development by targeting NDRG1. Moreover, xenograft model was widely used to analyze the pathogenesis of GC in vivo [28, 29]. Here we also established the murine xenograft model to validate the anti-cancer role of hsa_circ_0003159 and the regulatory network of hsa_circ_0003159/miR-223-3p/NDRG1 in vivo.

## Conclusion

In conclusion, this study disclosed the anti-cancer role of hsa_circ_0003159 in GC through inhibiting proliferation, migration, invasion and xenograft tumor growth and promoting apoptosis, possibly by regulating miR-223-3p and NDRG1. This research indicated a new target for treatment of GC.

## Supplementary information

**Additional file 1: Figure S1.** The effect of hsa_circ_0003159 on cell cycle in GC cells. The cell cycle distribution was detected in NUGC-3 and AGS cells transfected with vector or hsa_circ_0003159 by flow cytometry. *P < 0.05.

**Additional file 2: Figure S2.** The effect of hsa_circ_0003159 on epithelial-mesenchymal transition in GC cells. The expression levels of E-cadherin, N-cadherin, Snail and Slug were detected in NUGC-3 and AGS cells transfected with vector or hsa_circ_0003159 by western blot. *P < 0.05.

## Data Availability

The datasets used and/or analyzed during the current study are available from the corresponding author on reasonable request.
