# Supplementary figures and images for: Hsa_circ_0003159 inhibits gastric cancer progression by regulating miR-223-3p/NDRG1 axis
Source: Cancer Cell Int. 2020 Feb 19;20:57. doi: 10.1186/s12935-020-1119-0 (PMC7031989; doi:10.1186/s12935-020-1119-0)

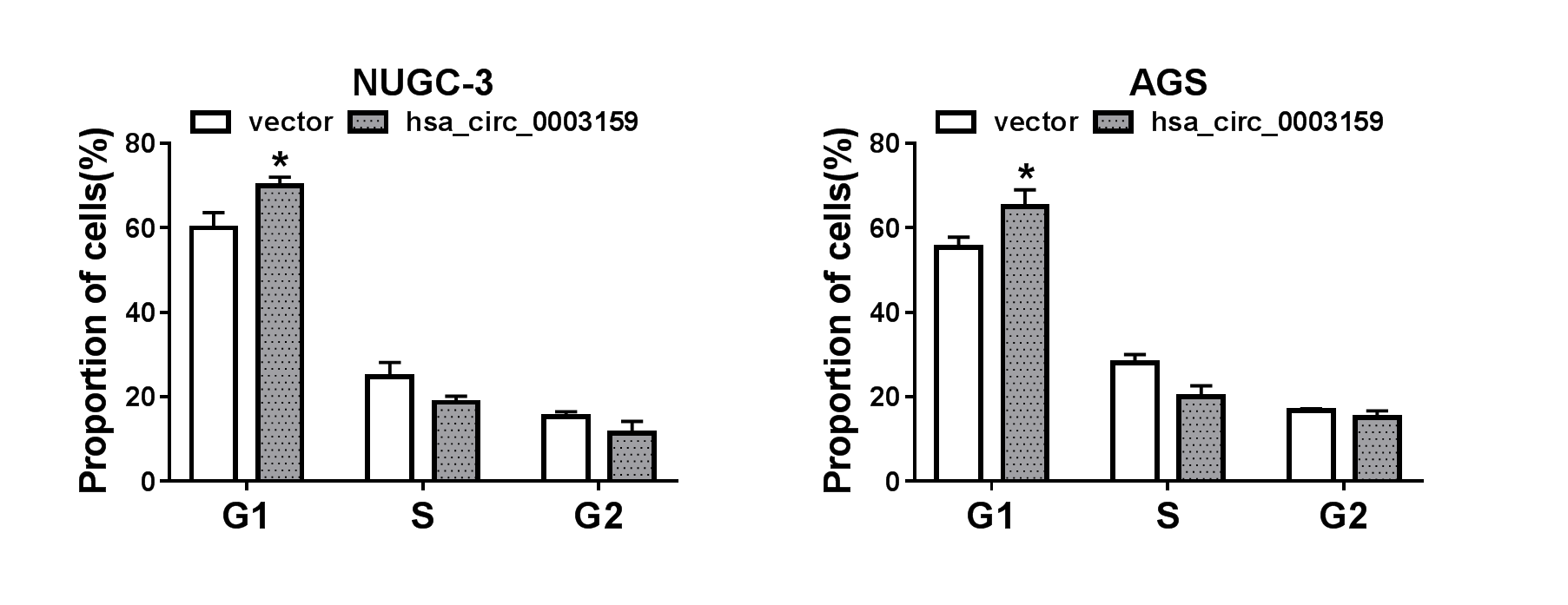

Supplement: Supplementary file 1 — Additional file 1: Figure S1. The effect of hsa_circ_0003159 on cell cycle in GC cells. The cell cycle distribution was detected in NUGC-3 and AGS cells transfected with vector or hsa_circ_0003159 by flow cytometry. *P < 0.05. [file 12935_2020_1119_MOESM1_ESM.tif]

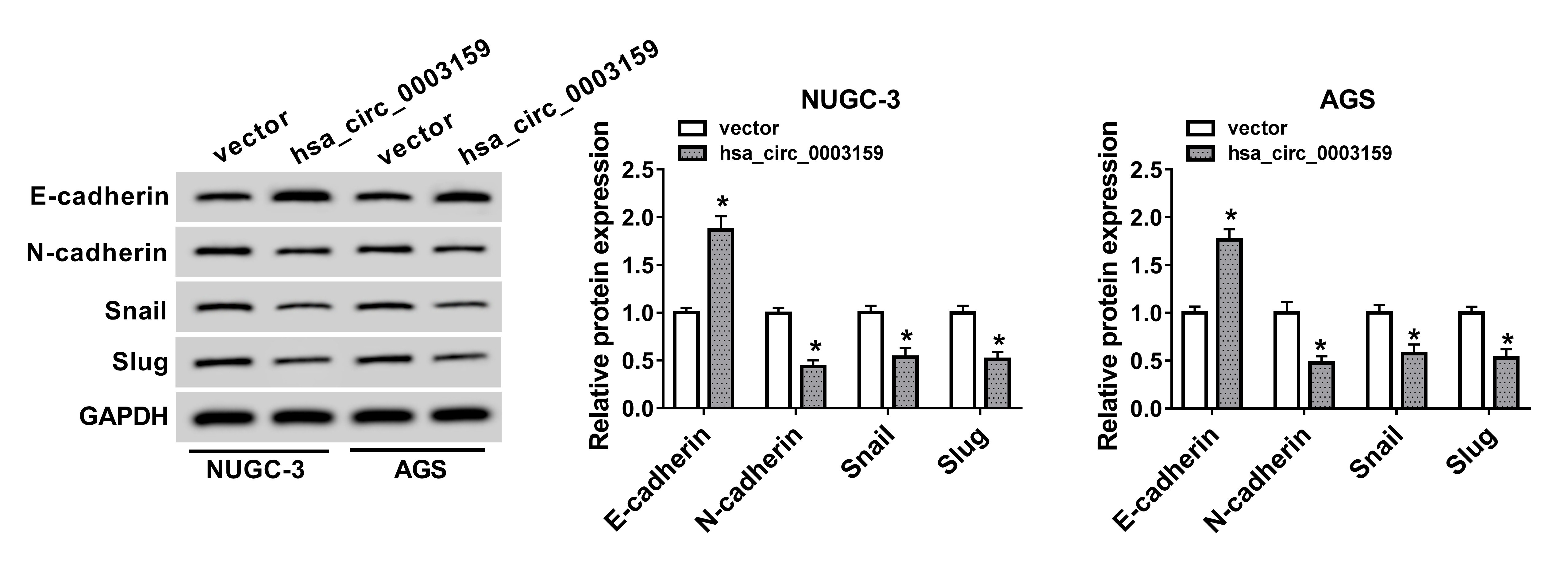

Supplement: Supplementary file 2 — Additional file 2: Figure S2. The effect of hsa_circ_0003159 on epithelial-mesenchymal transition in GC cells. The expression levels of E-cadherin, N-cadherin, Snail and Slug were detected in NUGC-3 and AGS cells transfected with vector or hsa_circ_0003159 by western blot. *P < 0.05. [file 12935_2020_1119_MOESM2_ESM.tif]
